# Urban change detection of remote sensing images via deep-feature extraction

**DOI:** 10.1038/s41598-025-07252-7

**Published:** 2025-07-01

**Authors:** Haiying Wang, Mingzhong Wu

**Affiliations:** 1 College of Earth Science, China West Normal University, Nanchong, 637000 China; 2 School of Mathematics and Information, China West Normal University, Nanchong, 637000 China

**Keywords:** Change detection, Deep learning, Bi-Unet, Dense block, LSTM, Ecology, Environmental social sciences

## Abstract

Urban change detection based on remote sensing images holds significant importance in environmental monitoring and emergency management. However, it poses several challenges including large disparity errors, diverse types of changes, and a substantial difference between the number of changed and unchanged areas. In this study, we propose an efficient model called BiUnet-Dense for extracting deep features by integrating the advantages of Bi-Unet, Dense Block, and long short-term memory (LSTM) networks. Building upon the classical architecture of U-Net, Bi-Unet utilizes bi-temporal images to compare and extract features. The incorporation of modified dense connections reduces network parameters while mitigating gradient disappearance through maximizing feature reuse. Additionally, LSTM facilitates information transmission from earlier to later using cell states to provide more meaningful feature vectors. We implemented our model on two datasets: Onera Satellite Change Detection (OSCD) and Change Detection Data of Guangzhou (CD_Data_GZ). Quantitative and qualitative results demonstrate that our method significantly improves detection effectiveness with enhanced F1-score and Kappa while effectively reducing false-positive detections as well as identifying labeling errors.

## Introduction

Change detection is a fundamental process that involves comparing multiple remote sensing images captured at different time points to identify alterations on Earth’s surface within the same geographical area. It serves as a crucial approach for acquiring up-to-date geographic information, making it a significant research focus in the field of remote sensing applications. The detection of land usage and land cover (LULC) changes can provide an efficient and cost-effective method for map updating, urban planning, and economic development prediction. Consequently, remote sensing studies have garnered considerable attention in recent years within the realm of remote sensing studies^1–4^.

The methods of change detection can be classified into two approaches: direct change prediction^5–7^ and first classifying and then identifying changes^8,9^. Furthermore, they can be categorized as classical machine learning-based methods^10,11^ or deep learning-based methods^12,13^. Initially, Walter performed pixel object classification in a GIS database to obtain change results^9^. Gu et al. proposed an enhanced Markov random field-based method for change detection^14^. Cao et al. extracted the changed and unchanged areas without relying on training parameters^15^. Lv et al. introduced a mixed conditional random field (mixed-CRF) model for change detection^11^. While classical machine learning-based change detection methods have demonstrated some success in practical applications, they exhibit limitations in terms of model generalization, assumptions about data distribution, computational complexity, interpretability of results, sensitivity to high-dimensional and noisy data, and dependence on labeled data. To address these challenges, researchers have increasingly turned to deep learning-based algorithms and techniques to enhance the accuracy and efficiency of change detection.

Change detection network architectures based on deep learning primarily encompass AlexNet^16^, Visual Geometry Group (VGG)^17^, Inception^18^, residual neural network (ResNet)^19^, fully convolutional network (FCN)^20^, U-Net^21^, and DeepLab V1 to DeepLab V3+^22–25^, which integrate components such as Inception, ResNet, and feature pyramid^26^. In the context of remote sensing multi-temporal urban change detection, Jiang et al. utilized a convolutional neural network to extract features from bi-temporal remote sensing images, connected these features sequentially, calculated the Euclidean distance of the feature maps, and finally obtained the change detection graph^12^. However, using Euclidean distance as a metric for change detection may be insufficiently sophisticated to fully capture complex spatial and temporal change information. Liu et al. employed an unsupervised deep convolutional coupling network to effectively detect changes between heterogeneous radar image datasets^27^. Although this method does not require labeled data, it is less accurate and comprehensive compared to supervised approaches in terms of change detection. Amirkolaee et al. estimated digital surface models (DSMs) using dense convolutional neural networks (DCNNs) to construct three-dimensional geospatial information based on single remote sensing images^28^, the accuracy of change detection using this method is contingent upon the precision of the DSMs. D’Addabboet calculated several deep features that efficiently captured contextual information for Very High-Resolution (VHR) image analysis using AlexNet-based pretrained convolution layers^29^. However, the processing of VHR images demanded substantial computational resources and time, thereby limiting the scalability of this method to large-scale datasets. Basavaraju et al. used modified residual connections and a new spatial pyramid pool module to complete change detection while preserving the shape of the changing region^30^. Although these improvements enhanced model performance, they also increased model complexity, leading to greater training difficulty and higher computational costs. Jinzhu et al. used the U-Net to capture historical urban development and simulate the future model of the North China Plain, which is the fastest urbanizing region in the world^31^. This approach focused primarily on rapid urbanization but was less sensitive to natural disasters, environmental changes, and other types of alterations. Iris et al. proposed a method that can directly perform change detection in 3D data. This method is a deep Siamese nuclear point convolutional network, which completes change detection and classification in one step through 2D image change detection and 3D point cloud analysis^32^. While this method achieved superior accuracy and improved classification performance, it also imposed high demands on computing resources and storage space. Overall, these studies demonstrated that deep learning methods exhibit superior accuracy, improved classification performance, and significantly reduced regional confusion.

To address the limitations of existing methods, capture complex features of remote sensing images, enhance change detection, improve model generalization and feature reuse capabilities, and capture long-term dependencies in time series, this study proposes an urban change detection method that integrates Bi-Unet, Dense Block, and LSTM. The main contributions are as follows:

1. Unlike the single encoder structure of traditional U-Net architectures, this model employs a Bi-Unet structure, incorporating an additional encoder to process inputs from two phases. By passing the difference matrix to the decoder for feature fusion, this approach better detects changes in images.
2. To capture complex features and enhance the model’s generalization and feature reuse abilities, dense connections are utilized in both the encoding and decoding units. However, these connections may increase network parameters, cause redundancy in backpropagation data, and raise GPU consumption during training. These issues are mitigated by adding a Dropout layer at the end of the dense connections.
3. In neural network backpropagation, layers closer to the current layer have a greater influence, while the influence of distant layers diminishes with increasing network depth. To capture the influence of distant layers, this model incorporates an LSTM network, leveraging gate control mechanisms to combine temporal features learned by different layers, thereby effectively capturing long-term dependencies in time series.

## Methods

**Fig. 1:**
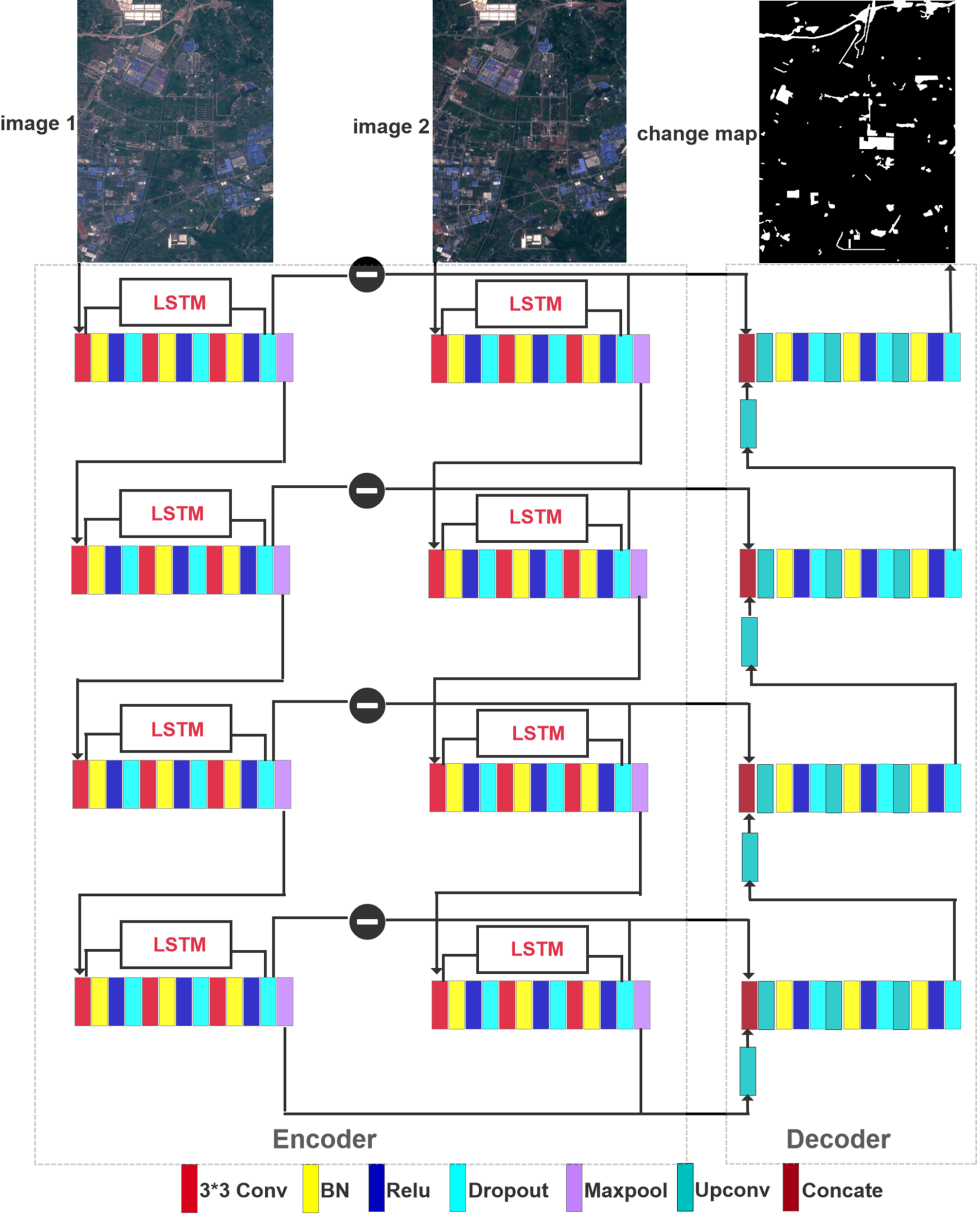
Architecture of the proposed BiUnet-Dense.

The proposed study introduces an urban change detection model based on the classical U-Net architecture, which enables more accurate semantic segmentation results with a reduced number of training samples. Figure 1 illustrates the specific architecture of our proposed model, which consists of an encoder and decoder. The encoder extracts low-level features from bi-temporal images, whereas the decoder extracts corresponding high-level features. The input to the model comprises bi-temporal images, and the output is the change map between them. The feature extraction of each image was performed in four units, with the detailed structures explained later in this paper. By calculating the differences between bi-temporal images, feature channels were added to establish a path for backward propagation during training that connects low-level features with their corresponding high-level counterparts^21^. The four skip connections connect the four decoder units. To ensure feature reuse across each unit, both the encoder and decoder adopted Dense Blocks to connect feature maps throughout the network layer. LSTM networks are employed for each block in low-level feature extraction to address long-term dependencies in simple recurrent neural networks (RNNs).

### Bi-Unet

**Fig. 2:**
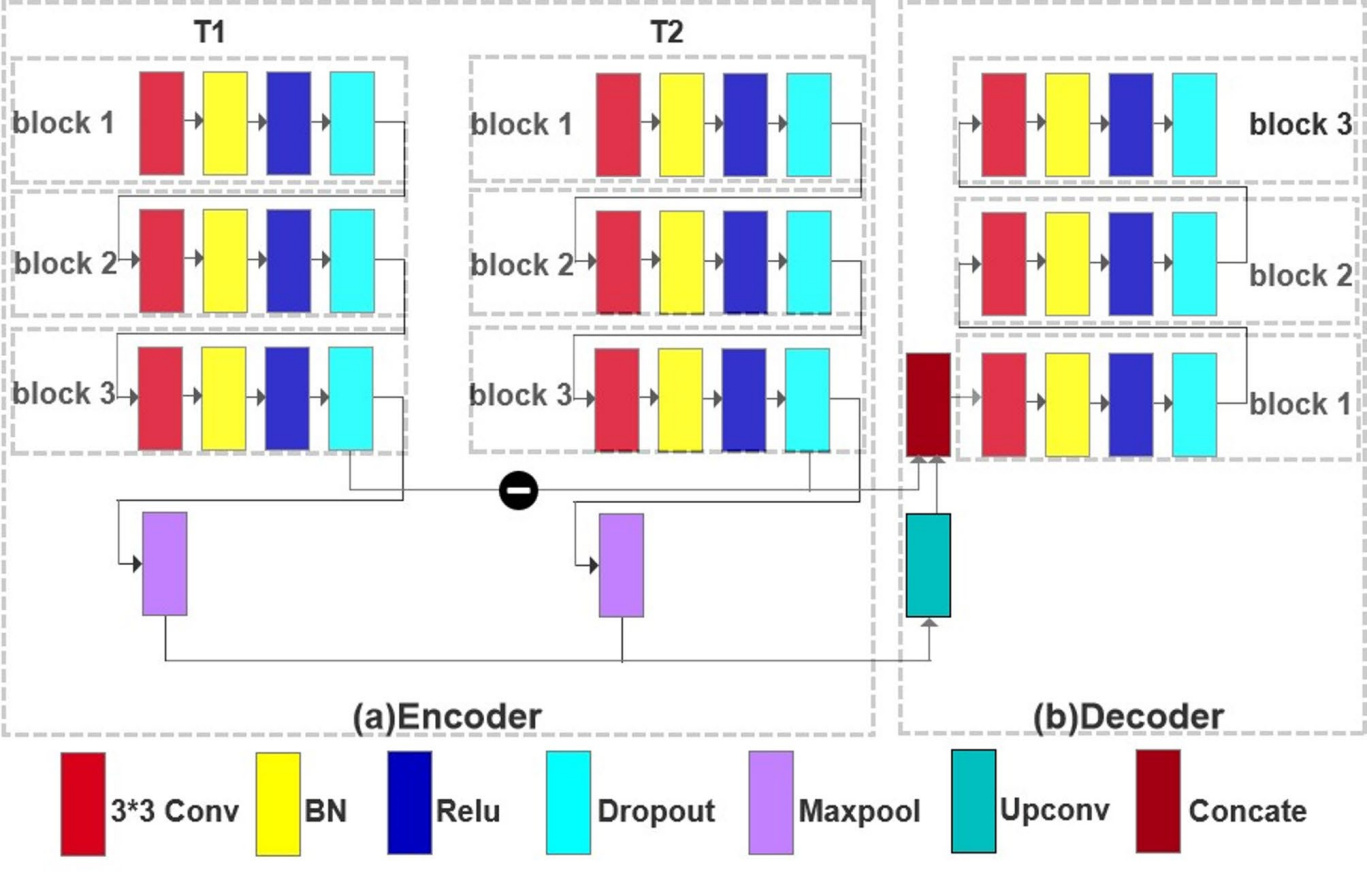
The specific modules unit of both the encoder and decoder.

In recent years, the proliferation of deep learning-based methodologies across diverse domains has led to a plethora of change detection techniques. In^33,34^, a novel deep patch-based architecture wherein features extracted from bi-temporal patches are simultaneously processed through an array of expanded convolutional layers. However, this approach requires pixel-level processing at the individual level, which results in computationally intensive operations. Daudt et al. introduced three distinct fully convolutional Siamese networks for change-region detection^35^; however, their models lacked sufficient consideration of temporal data patterns. To address these limitations, we propose a deep learning model based on Bi-Unet, which effectively captures spatial features from bi-temporal inputs. Figure 2 illustrates the specific modules in both the encoder and decoder units.

The architecture of Bi-Unet is based on the U-Net network. The core feature of the U-Net network lies in its distinctive U-shaped structure, which consists of two main components: an encoder and a decoder. In the encoder section, image features are progressively extracted via multi-layer convolutional and pooling operations, thereby reducing the data dimensionality. In the decoder section, the spatial dimensions and details of the image are gradually restored through upsampling operations and skip connections. Skip connections, a key characteristic of the U-Net network, facilitate the effective fusion of features at different scales by concatenating the feature maps from the encoder with the upsampled results from the decoder. This design enables the U-Net network to capture fine structures within images, thereby enhancing the segmentation accuracy for small targets and edge regions. The U-Net network employs a fully convolutional architecture, eliminating the need for fully connected layers found in traditional CNNs, thus reducing the number of parameters and computational load. Additionally, this architecture allows the U-Net network to process input images of any size, improving the model’s flexibility and practicality. Building upon the single-encoder structure of the traditional U-Net architecture, Bi-Unet introduces an additional encoder to receive information from two phases, passing the difference matrix to the decoder for feature fusion, thereby better detecting changes in the image.

As illustrated in Fig. 2, the proposed model incorporates the bi-temporal inputs of T1 and T2. Each encoder unit consists of three blocks, each comprising a 3 × 3 convolutional layer, batch normalization (BN) layer, rectified linear unit (ReLU) layer, and Dropout layer. Subsequent to the feature extraction process in the encoder, the extracted feature maps from the bi-temporal images were down-sampled through 2 × 2 max-pooling. During the decoder phase, the max-pooling results undergo transposed convolution before computing the difference between them and those obtained from block bi- temporal images in the encoder. This difference was concatenated with the transposed convolution results to serve as an input for block1. By incorporating skip connections within the Bi-Unet architecture, high-resolution information is integrated with low-resolution information to generate more intricate features while preserving the spatial and temporal details. Each decoder unit comprises three blocks consisting of a 3 × 3 convolutional layer, BN layer, ReLU layer, and Dropout layer. Finally, a final urban change map was derived at the end of our model using a 1 × 1 convolutional layer. The output of the model represents the changes observed between bi-temporal images.

### Dense block

Since the introduction of ResNet^19^, two prominent trends have emerged in the backbone network structures: increased depth and width. Huang et al. proposed a technique called stochastic depth to train networks by randomly eliminating layers while ensuring convergence of the algorithm^36^. To address issues pertaining to network redundancy, Huang et al. introduced a novel concept based on the stochastic depth of ResNet, known as the DenseNet^37^. It builds upon the stochastic depth concept of ResNet. The core component of DenseNet is the Dense Block, whose primary objectives are to mitigate vanishing gradients, significantly reduce parameter count, enhance feature extraction, and promote feature reuse while maintaining a compact model size. In a Dense Block, multiple convolutional layers are densely connected, with each layer receiving input from all preceding layers. Specifically, for an N-layer Dense Block, the Nth layer’s input consists of concatenated feature maps from all previous N-1 layers along the channel dimension. This design facilitates smoother information flow and more efficient feature reuse. As the number of layers increases, the number of input feature maps for each layer grows accordingly. In an N-layered network, N*(*N* + 1)/2 connections facilitate seamless information flow and gradient propagation throughout the entire network structure. This characteristic not only simplifies the training, but also enhances the feature extraction performance.

**Fig. 3:**
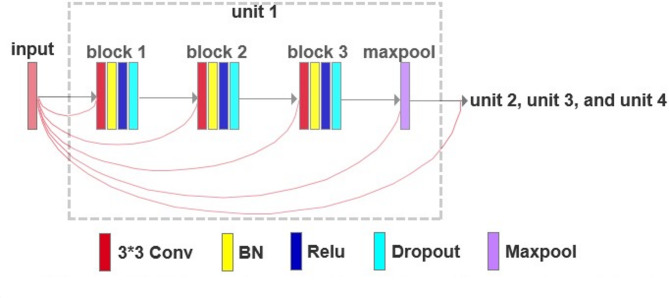
The modified dense interconnections between the encoder and decoder.

The dense interconnections between the encoded and decoded units are shown in Fig. 3. Specifically, the input image is connected to block1, block2, block3, a max pooling layer, and subsequent units. Assume that the input image is denoted as ${x}_{0}$, the output of layer $l$ is represented by ${x}_{l}$, and the transformation performed by each dense convolutional block is denoted as ${H}_{l}\left(\right)$, where $l$ denotes the layer number. In a conventional feedforward neural network setting, we have ${x}_{l}={H}_{l}\left({x}_{l-1}\right)$. However, in ResNet, the equation becomes ${x}_{l}={H}_{l}\left({x}_{l-1}\right)+{x}_{l-1}$, whereas in DenseNet, it becomes ${x}_{l}={H}_{l}\left(\left[{x}_{0},{x}_{1},\dots\:\dots\:,{x}_{l-1}\right]\right)$, where [·] denotes the concatenation operation. In our proposed model design approach, we specifically employ ${H}_{l}\left(\right)$ consisting of a 3 × 3 convolutional operations, followed by a BN and ReLU activation function application. Due to the use of Dense Blocks, as the number of layers increases, the number of input feature maps gradually grows, leading to a significant increase in computational complexity per layer. This connection mode may result in issues such as an increase in network parameters, redundant backpropagation data, and higher GPU consumption during training. To address these challenges, we introduce a Dropout layer at the end of the dense connections, as depicted in Fig. 3. The Dropout layer randomly sets a subset of neurons’ outputs to zero, thereby reducing the computational load during training and mitigating neuron coadaptation, which enhances the model’s generalization ability and prevents overfitting.

### LSTM block

The encoding unit employs a recurrent network (RNN) for feature map extraction. RNNs^38^ have been widely adopted for handling time-dependent data because of them ability to capture temporal relationships among sequential features. LSTM^39^, a sophisticated recurrent module comprising four interconnected neural networks, has been widely applied in diverse domains, such as motion trajectory prediction^40–42^ and movement-related time-series forecasting^43–45^. By incorporating four interacting layers to address long-term dependency issues, LSTM stands out among other types of RNNs. The gates of LSTM selectively incorporate or exclude information from the cell state through activation functions, thereby enabling precise control over the flow of information. Each step of the LSTM processing, an output denoted as h and a cell state represented by C are generated. The specific operations of LSTM can be summarized as follows:

To ascertain the information that can be transmitted through the cellular state for sequence X and input vector ${x}_{t}$ at time t, a pivotal initial step involves making a decision regulated by the forget gate utilizing the sigmoid activation function, as illustrated in (1).

1 $${f}_{t}={\upsigma\:}({W}_{f} \cdot \left[{h}_{t-1},{x}_{t}\right]+{b}_{f}).$$

where the sigmoid function ${\upsigma\:}$ is utilized with the weight matrix ${W}_{f}\:$employed by the forget gate, ${h}_{t-1}$ represents the previous output, ${x}_{t}$ denotes the current input, and ${b}_{f}$ indicates the bias. Equation (1) constrains the value of$\:{f}_{t}$ between 0 and 1, thereby determining the extent to which ${C}_{t-1}$ can be propagated.

The second step involves generating new information that requires updating and consists of two components. First, the input gate determines the values to be updated using a sigmoid activation function, as shown in (2). Second, a novel candidate value, ${\stackrel{\sim}{C}}_{t}$ is generated by the tanh function, as shown in (3). This candidate value can be incorporated into the cell state that is generated by the current layer.

2 $${i}_{t}={\upsigma\:}({W}_{i} \cdot \left[{h}_{t-1},{x}_{t}\right]+{b}_{i})$$

3 $${\stackrel{\sim}{C}}_{t}=tanh({W}_{c} \cdot \left[{h}_{t-1},{x}_{t}\right]+{b}_{c}).$$

where the sigmoid function$\:{\upsigma\:}$ and hyperbolic tangent function tanh are utilized, ${W}_{i}$ and ${W}_{c}$ represent the corresponding weight matrices, ${h}_{t-1}$ denotes the previous output, ${x}_{t}$ represents the current input, and ${b}_{i}$ and ${b}_{c}$ indicate the bias. Equation (4) was employed to combine the outputs from these two components to facilitate the cell state update.

4 $${C}_{t}={f}_{t}\text{*}{C}_{t-1}+{i}_{t}\text{*}{\stackrel{\sim}{C}}_{t}.$$

The final step involved determining the output of the model. Initially, the output was obtained by utilizing the output gate, as shown in (5). Subsequently, the tanh function was employed to rescale ${C}_{t}$ within the range of −1 to 1. This rescaled value is then multiplied element-wise by the output from (5), as shown in (6). Ultimately, ${h}_{t}$ represents the output of the LSTM.

5 $${o}_{t}={\upsigma\:}({W}_{0} \cdot \left[{h}_{t-1},{x}_{t}\right]+{b}_{o})$$

6 $${h}_{t}={o}_{t}{*}\text{t}\text{a}\text{n}\text{h}\left({C}_{t}\right).$$

**Fig. 4:**
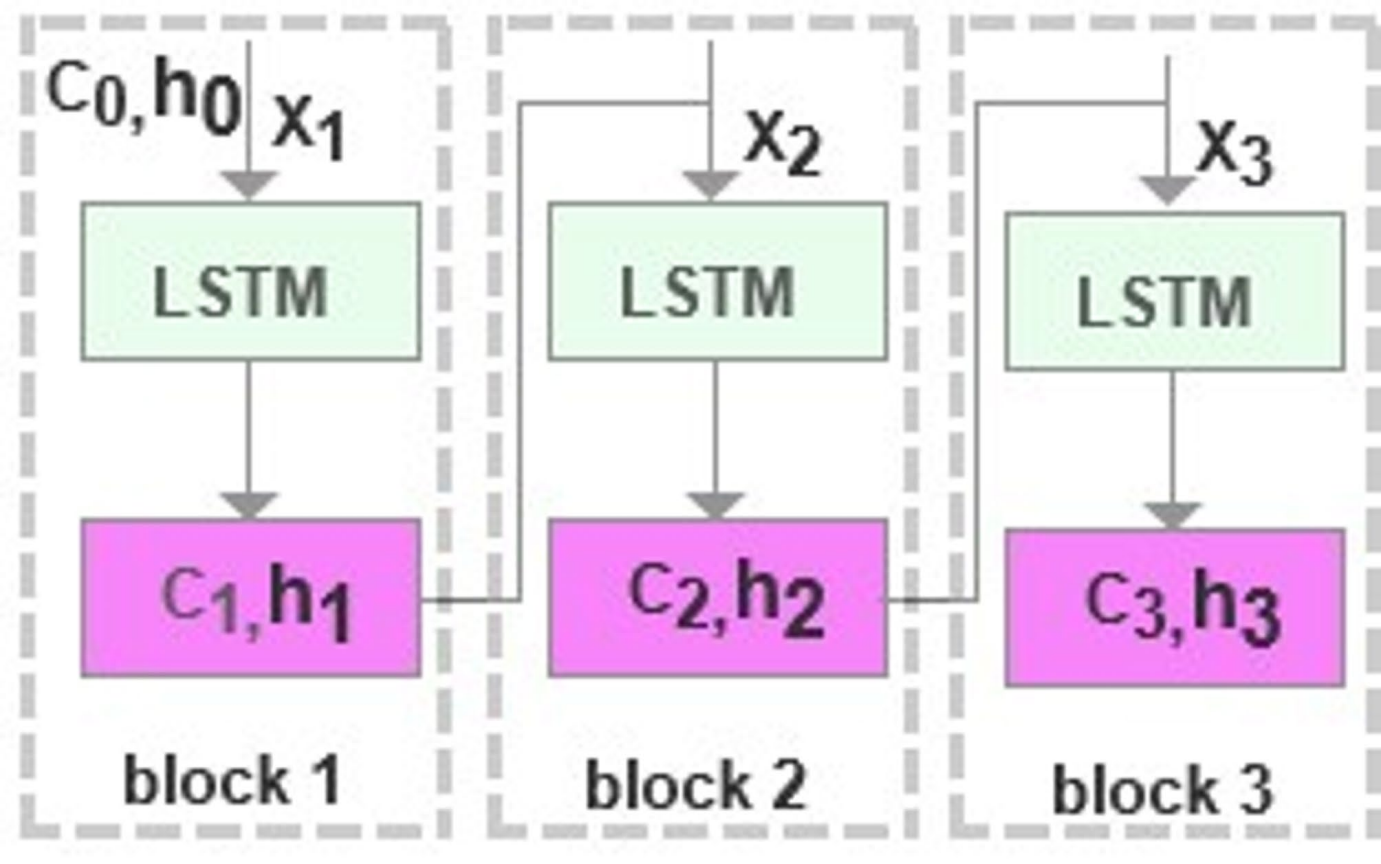
The loop blocks of LSTM layer.

The detailed processing steps of the three LSTM layer loop blocks within a single unit at any given time in the encoder are shown in Fig. 4. Each unit consists of three blocks, where each block receives a vector representation based on the time series as the input and is connected to the output of the preceding block. Block 1 takes $[{C}_{0},{h}_{0}]$ and ${x}_{1}$ as inputs and generates $[{C}_{1},{h}_{1}]$ as its output. Similarly, block 2 uses $[{C}_{1},{h}_{1}]$ and ${x}_{2}$ as inputs, yielding $[{C}_{2},{h}_{2}]$ as its output.

As depicted in Fig. 1, the input of the encoder for any bi-temporal sequence comprises five units, with each output serving as the input of the subsequent unit. Given that this model consists of multiple LSTM layers, the first layer receives the initial input data, whereas each successive layer uses the output of the predecessor as its own input. Ultimately, the final output vector corresponds to the final step of the last layer. Within this network architecture, a single vector representation encodes information regarding all geographical locations within the cell state. Consequently, although initially distinct across different input channels, their unique characteristics are subsequently blended into an LSTM cell-state vector and utilized as the final output.

### Change detection

The proposed model presents a network architecture based on Bi-Unet that incorporates two streams of the same shared weights for the encoder. These streams are assigned to input bi-temporal images to enable more accurate change detection. To facilitate skip connections, the absolute difference between bi-temporal images was utilized. Each unit in both the encoder and decoder consisted of three blocks. To address potential feature overwriting and resolve long-term dependence issues in RNNs, LSTM networks were employed within each unit of the encoder. Furthermore, Dense Blocks are incorporated in both the encoder and decoder to enhance the feature transmission and extract additional change information. Specifically, each layer takes as input not only from all previous layers, but also ensures that subsequent layers can effectively utilize the extracted features.

In our model, the spatial details present in the early layers can complement the more abstract and comparative information encoded later, thereby leading to enhanced accuracy in predicting boundary changes in the output image. Importantly, we incorporated change detection or transfer learning from other datasets, enabling end-to-end training of the architectures. The OSCD and CD_Data_GZ datasets were used as the experimental data in this study. The input and output images were preserved with their original dimensions; the parameters of each block are listed in Table 1.

**Table 1:** Network structure of BiUnet-Dense.

	Time	Block	Conv layer	Filter	Outputsize
Encoder	T1	block1	con11–con13	[3 × 3,16]×3	[16,35,3,3]
		block2	con21–con23	[3 × 3,32]×3	[32,115,3,3]
		block3	con31–con33	[3 × 3,64]×3	[64,275,3,3]
		block4	con41–con43	[3 × 3,128]×3	[128,595,3,3]
	T2	block1	con11–con13	[3 × 3,16]×3	[16,35,3,3]
		block2	con21–con23	[3 × 3,32]×3	[32,115,3,3]
		block3	con31–con33	[3 × 3,64]×3	[64,275,3,3]
		block4	con41–con43	[3 × 3,128]×3	[128,595,3,3]
Decoder		block1	con43d–con41d	[3 × 3,128]×2 [3 × 3,64]×1	[512,64,3,3]
		block2	con33d–con31d	[3 × 3,64]×2 [3 × 3,32]×1	[256,32,3,3]
		block3	con23d–con21d	[3 × 3,32]×2 [3 × 3,16]×1	[128,16,3,3]
		block4	con13d–con11d	[3 × 3,16]×2 [3 × 3,2]×1	[16,2,3,3]

## Results

### Implementation details

We implemented BiUnet-Dense using the nn.Module in the PyTorch framework. All images were utilized without preprocessing and were inputted to their original dimensions. To augment the training sample diversity, shape adjustment, random flipping, and random rotation were incorporated into a size of 32 × 32 during each iteration. The hyperparameters were set as follows: 50 epochs, a batch size of 32, and a patch size of 96. The model was trained using an NVIDIA GeForce GTX 2080Ti and Intel(R) Xeon(R) CPU E5-2690 v4.

### Dataset description

The OSCD dataset addresses the challenge of detecting temporal variations in multispectral satellite images captured by Sentinel-2 between 2015 and 2018^46^. This dataset comprises 24 pairs of carefully selected multispectral image sets acquired from diverse locations worldwide, including Brazil, the USA, Europe, the Middle East, and Asia. Each location was represented by a set of multispectral images consisting of 13 bands obtained from the Sentinel-2 satellite. The spatial resolution ranges from 10 m to 60 m.

The CD_Data_GZ dataset was collected from 2006 to 2019, covering the suburban areas of Guangzhou, China^47^. To facilitate the generation of image pairs, we utilized the Google Earth service within BIGEMAP to acquire 19 seasonal VHR image pairs with three spectral bands: red, green, and blue. This dataset has a spatial resolution of 0.55 m and dimensions ranging from 1006 × 1168 pixels to 4936 × 5224 pixels.

### Quantitative evaluation metrics

To evaluate the performance of our proposed urban change detection algorithm and compare it with other methods, we employed a set of evaluation metrics. Prior to conducting an in-depth analysis of these metrics, it is necessary to define fundamental terminology. TP, FP, TN, and FN represent the numbers of true positives, false positives, true negatives, and false negatives, respectively. In the change detection module, TP refers to the accurately identified amount of data indicating change, whereas FP represents instances incorrectly classified as changed. TN represents accurately classified data indicating no change, whereas FN signifies erroneously identified data indicating no change. The quantitative evaluation metrics included precision, recall, F1-score, and the Kappa index. Precision quantifies the ability to avoid false detections by dividing the number of true positives by the total number of instances and is defined as follows:

7 $$Precision=\frac{TP}{TP+FP}.$$

Recall denotes the ratio of true positives to the total number of positive instances and serves as a metric for effectively minimizing false negatives. This is mathematically defined as follows:

8 $$Recall=\frac{TP}{TP+FN}.$$

The precision and recall metrics exhibited contrasting behaviors, indicating an inverse relationship between recall and precision. Conversely, an increase in recall results in a decrease in precision and vice versa. The F1-score is defined as the harmonic mean of recall and precision, rendering it a more comprehensive measure of the algorithm performance. This can be regarded as a descriptive indicator that encompasses both recall and precision.

9 $$F1-score=\frac{2\times\:Precision\times\:Recall}{Precision+Recall}.$$

The Kappa index^48^ is a statistical measure used to evaluate the level of agreement among raters in classifying the results. It is widely recognized as a more robust metric than a simple calculation of agreement percentage, because it accounts for the possibility of chance agreement. The Kappa index ranged from 0 to 1, with higher values indicating superior performance of the classifier. By considering both the observed relative agreement between the classifier and ground truth ${p}_{o}$ and the hypothetical probability of chance agreement ${p}_{e}$, the Kappa index can be defined as:

10 $$Kappa=\frac{{p}_{o}-{p}_{e}}{1-{p}_{e}}.$$

where the ${p}_{o}$ and the ${p}_{e}$ are given by

11 $${p}_{o}=\frac{TP+TN}{TP+FP+TN+FN}$$

12 $${p}_{e}=\frac{\left(TP+FP\right)\left(TP+FN\right)+\left(TN+FP\right)(TN+FN)}{{(TP+FP+TN+FN)}^{2}}.$$

## Experimental results

To evaluate the performance of BiUnet-Dense, we conducted a comparative analysis against some bi-temporal change detection methods, including fully convolutional Siamese concatenation (FC-Siam-conc)^35^, dual-task constrained deep Siamese convolutional network (DTCDSCN)^49^, the combination of Siamese network and NestedUNet (SNUNet)^50^, bitemporal image transformer (BIT)^51^, attention-based multiscale transformer network (AMTNet)^52^, and transformer-based Siamese two-stream CD framework (ScratchFormer)^53^. To ensure consistency, we implemented all five networks using the PyTorch framework and trained them on both datasets.

### Quantitative results

Table 2 presents the parameters and four evaluation metrics for urban change detection in BiUnet-Dense along with the comparison bi-temporal methods. Precision and recall cover both the changed and unchanged features. The results show that our method performs well in multiple aspects.

It can be seen from Table 2 that in the models of comparative analysis, BiUnet-Dense demonstrates outstanding performance on both the OSCD and CD_Data_GZ datasets, ranking first in both F1 score and Kappa coefficient. Compared to FC-Siam-conc, DTCDSCN, SNUNet, BIT, AMTNet, and ScratchFormer in the OSCD dataset, BiUnet-Dense demonstrated a significant improvement in the F1-score by 19.54%, 16.44%, 16.26%, 15.16%, 15.65%, and 0.28%, respectively, while its Kappa increased by 19.56%, 17.31%, 17.03%, 15.25%, 15.45%, and 0.23%, respectively. On the CD_Data_GZ dataset, compared with the comparison models, the F1-score of BiUnet-Dense increased by 2.46%, 0.51%, 9.04%, 0.42%, 4.13%, and 0.64%, respectively, and the kappa increased by 1.56%, 11.03%, 9.52%, 3.1%, 2.56%, and 1.26%, respectively. Especially in the OSCD dataset, it performs outstandingly in the precision and recall of change categories, but its parameters and computational complexity are relatively large. ScratchFormer and BIT follow closely behind. The former strikes a balance between performance and computational load, while the latter performs exceptionally well in terms of the accuracy rate of change categories. DTCDSCN and SNUNet have high computational efficiency and are suitable for resource-constrained scenarios, but their performance is compromised. AMTNet and FC-Siam-conc each have their own advantages in different indicators. Therefore, in the case of increasingly abundant computer resources, BiUnet-Dense is the first choice for pursuing ultimate performance.

**Table 2:** Comparison results on the two change detection datasets.

Models	Params (M)	FLOPs (G)	OSCD Pre_c/Rec_c/Pre_nc/Rec_nc/F1/Kappa	CD_Data_GZ Pre_c/Rec_c/Pre_nc/Rec_nc/F1/Kappa
FC-Siam-conc	15.46	50.77	35.29/25.59/96.18/97.56/29.67/26.62	**69.06**/45.25/93.24/**97.38**/54.68/50.18
DTCDSCN	41.07	7.21	33.96/29.05/92.60/94.65/32.77/28.87	60.97/55.09/**97.76**/85.45/56.63/40.71
SNUNet	**12.03**	27.44	34.21/27.64/93.28/95.02/32.95/29.15	67.46/50.58/90.98/93.85/48.10/42.22
BIT	26.30	68.50	35.73/29.79/**97.95**/95.63/34.05/30.93	63.09/51.23/91.98/91.40/56.72/48.64
AMTNet	16.45	**14.80**	34.76/29.66/95.83/97.01/33.56/30.73	57.25/55.63/90.69/94.97/53.01/49.18
ScratchFormer	55.58	48.79	39.22/49.90/96.04/**97.78**/48.93/45.95	56.29/**56.14**/89.41/96.21/56.50/50.48
BiUnet-Dense	43.10	53.54	**43.22**/**57.13**/97.93/96.09/**49.21**/**46.18**	58.29/56.03/94.36/94.83/**57.14**/**51.74**

**Fig. 5:**
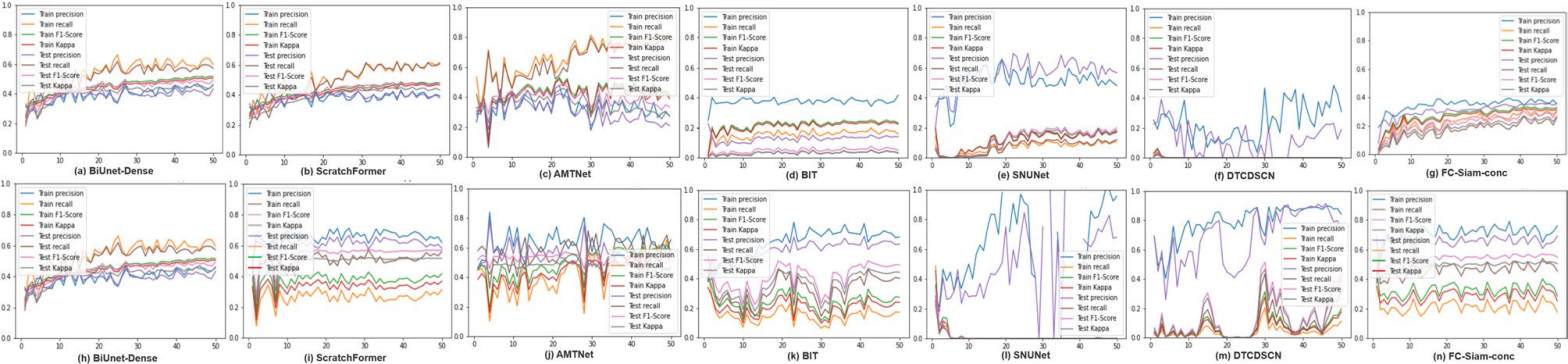
The evaluation matrices for the training and texting phases of the both datasets.

The evaluation matrices of all models in the training and testing phases are shown in Fig. 5, where (a)–(g) are the evaluation matrices on the OSCD dataset, and (h)–(n) are the evaluation matrices on the CD_Data_GZ dataset. It can be seen from the first row of Fig. 5 that on the 0SCD dataset, compared with BiUnet-Dense, although the precision, recall, F1-scoreh and Kappa of ScratchFormer are very close to the metrics on BiUnet-Dense, However, after the model training reaches the stable stage, there is still a problem in ScratchFormer that the precision and F1-score in the test stage are higher than those in the training stage. This overfitting performance can imply that the model has not fully learned the features of the training set. Or the regularization method was wrongly applied in the testing phase, resulting in the model performing better during the test. The longer the AMTNet model is trained, the more it shows a trend of being unfitting. The evaluation indicators of several models such as BIT, SNUNet, and FC-Siam-conc are relatively low. The DTCDSCN model not only has very low evaluation index values but also oscillates severely. Evidently, BiUnet-Dense consistently outperformed the other models in both the training and testing phases. In the training phase, precision, recall, F1-score, and Kappa were reported as 43.52%, 60.67%, 50.68%, and 49.53%, respectively, whereas in the testing phase, they were recorded as 41.98%, 54.29%, 47.34%, and 43.32%, respectively. Furthermore, the results from BiUnet-Dense exhibited smoother and more stable performance with similar growth rates across both the training and testing phases without notable discrepancies.

On the CD_Data_GZ dataset, compared with BiUnet-Dense, the precision, recall, F1-score, and Kappa of ScratchFormer, BIT, and FC-Siam-conc during the training stage were significantly higher than those on BiUnet-Dense. However, the values of these four indicators were very low during the testing stage of these models. It can be seen that overfitting has occurred in these three models. The remaining three models, AMTNet, SNUNet, and DTCDSCN, have been oscillating severely all the time, making the training impossible to fit. Therefore, BiUnet-Dense outperformed the other two models on both the train and test datasets. During the training phase, precision, recall, F1-score, and Kappa achieved values of 75.32%, 43.93%, 55.49%, and 43.97%, respectively. During the testing phase, these values were recorded as 57.98%, 33.64%, 42.56%, and 38.91%, respectively.

### Qualitative results

**Fig. 6:**
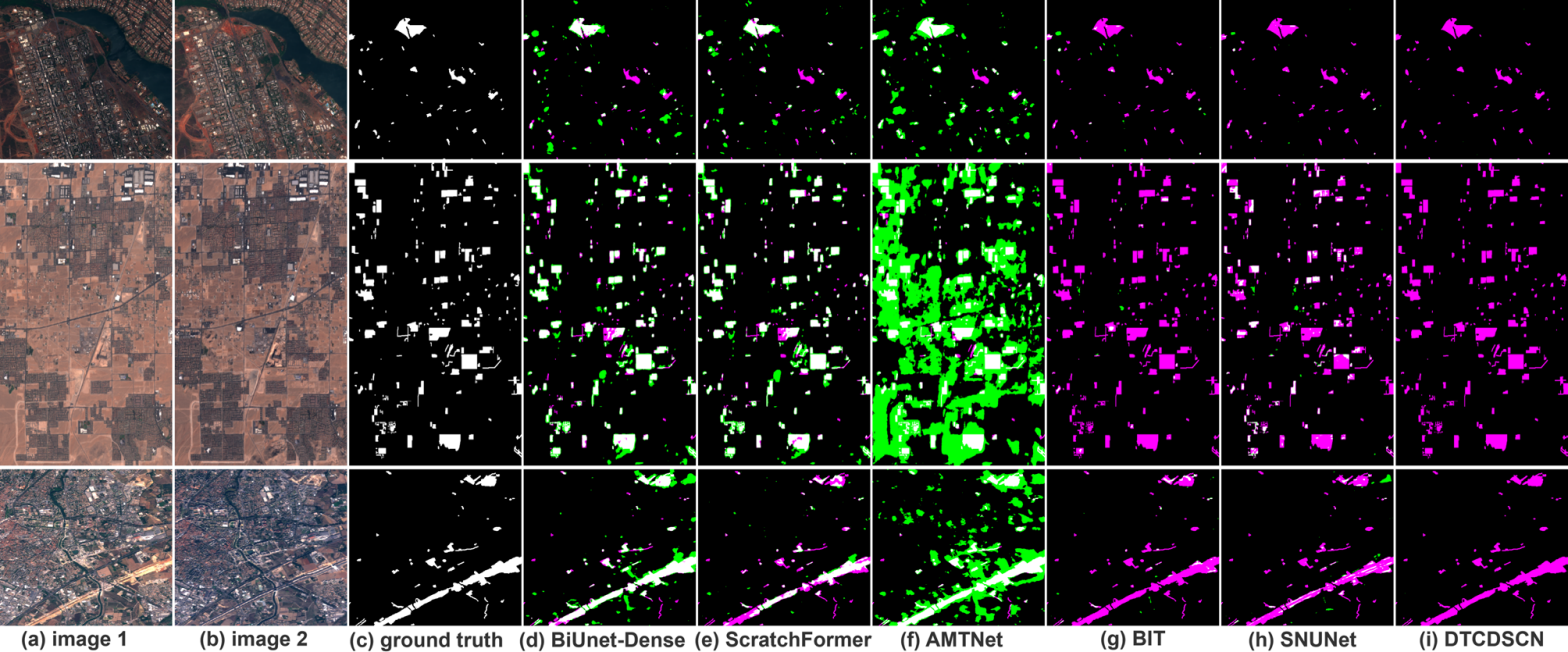
Change detection from the OSCD dataset using the different methods on different scences.

Figure 6 shows the change detection outcomes of the various methods utilizing the three channels on the OSCD dataset. The OSCD dataset demonstrates a low resolution, with the ground truth marking all relative changes between the two time periods, encompassing modifications in buildings, roads, vegetation, water bodies, and other features. In this figure, white, black, green, and magenta represent TPs, TNs, FPs, and FNs, respectively. These results clearly indicate that all methods are influenced by variations in lighting conditions, as well as the spatial characteristics of buildings, such as rooftops. Moreover, they are affected by alterations in vegetation cover, bare soil, and water bodies. BiUnet-Dense exhibited limitations in accurately delineating boundaries, leading to the presence of numerous FP instances surrounding the predicted TPs. However, BiUnet-Dense demonstrated superior performance by detecting a greater number of TPs while maintaining high levels of accuracy compared to other methods. Although some FPs persist within their results, their frequency is lower than that observed with alternative approaches and is primarily concentrated around the predicted TPs. Furthermore, BiUnet-Dense exhibits fewer FN predictions than the other detection techniques employed herein. Notably, resilient against unrelated urbanization changes, it successfully identified a substantial number of alterations occurring within buildings and roads. Although ScratchFormer and AMTNet also correctly identified most of the TPs, compared with BiUnet-Dense, there are more FNs in the detection results of ScratchFormer, and there are many FPs around the TPs detected by AMTNet. This might be due to the problem of local feature dilution in the global context modeling of the transformer architecture of ScratchFormer, which leads to the accumulation of FNs. Although AMTNet enhances semantic association, in dense prediction scenarios, the cross-attention branches it designs may introduce feature pollution, causing the spread of FPs. The remaining three models, BIT, SNUNet and DTCDSCN, adopted multi-scale feature fusion or edge enhancement modules, thus achieving excellent results in the aspect of change detection boundaries. However, almost all of them identified all the true examples as false counterexamples.

**Fig. 7:**
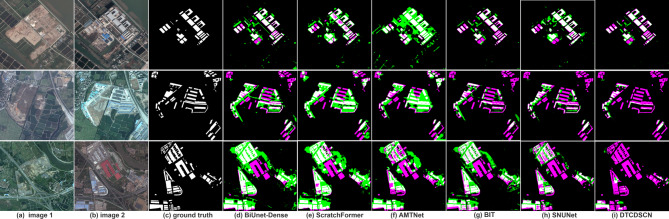
Change detection from the CD_Data_GZ dataset using the different methods on different scences.

The change detection results of the different methods on the CD_Data_GZ dataset are presented in Fig. 7. Because the dataset exhibited a higher resolution, with ground-truth annotations limited to building changes only. All methods demonstrated precise boundary delineation in their prediction results. Particularly, BiUnet-Dense performs better in the detection of continuous and dense fine changes. Almost all models wrongly classify the dark building roofs resulting from changes as FNs. The models overly rely on the characteristics of the surrounding environment. When the dark roof is in a heterogeneous area, the context semantic bias will suppress the change detection. Among these approaches, BiUnet-Dense achieved superior performance by identifying the highest number of TPs and detecting subtle changes in both buildings and roads that were not annotated in the ground truth data. Consequently, all roads identified by BiUnet-Dense were marked as FPs because of their absence from the provided annotations. In urban change detection tasks, our primary focus is to capture changes, specifically in buildings and roads. Although some unrelated changes may be detected as FPs, BiUnet-Dense demonstrates greater robustness towards urbanization-related alterations.

## Discussion

### Evaluation of the Bi-Unet

The dual-encoder architecture employed by Bi-Unet provides two parallel feature extraction pathways for U-Net networks. Each pathway independently processes the input image, extracting distinct feature sets and computing their differences to generate a difference matrix. This parallel processing approach enables the model to capture more diverse image information, thereby enhancing the diversity of feature extraction. Compared with single-encoder U-Net networks, the dual-encoder architecture facilitates the formation of multiple feature representations during the encoding process. Each encoder branch can learn unique feature maps that are effectively utilized to recover image details and delineate boundaries during decoding. By integrating these diverse feature representations, the model can produce more accurate and detailed segmentation results. Additionally, the introduction of the dual-encoder structure enhances the model’s generalization capability. Since the two encoder branches extract features independently, which are subsequently fused and leveraged in later network layers, the model demonstrates improved adaptability to various types of images and segmentation tasks. Consequently, this design allows the model to perform robustly even when processing unseen images.

As shown in Table 3, the application of the dual-encoder model resulted in significant improvements in the OSCD dataset, with precision_change, recall_change, precision_no_change, F1_score, and Kappa increasing by 8.61%, 31.52%, 1.55%, 19.77%, and 20.05%, respectively. On the CD_Data_GZ dataset, these metrics improved by 1.55%, 3.16%, 0.38%, 2.4%, and 3.38%, respectively. These enhancements can be attributed to the diverse feature representations generated by the dual-encoder, leading to more accurate and detailed segmentation results. In urban change detection tasks, the unchanged areas typically constitute a larger proportion compared to changed areas, potentially causing an imbalance between positive and negative samples. The introduction of dual encoders may have exacerbated this imbalance by extracting features from multiple dimensions, thereby focusing more on changed regions and less on unchanged regions. Consequently, the recall_no_change metric decreased by 1.39%.

**Table 3:** Evaluation results of architectures with dual-encoder and single-encoder.

Models	Dataset	Pre_c (%)	Rec_c (%)	Pre_nc (%)	Rec_nc (%)	F1 (%)	Kappa (%)
Method with dual-encoder	OSCD CD_Data_GZ	43.22 **58.29**	**57.13** 56.03	**97.73** 94.36	96.09 94.83	49.21 **57.14**	46.18 **51.74**
Method with single-encoder	OSCD CD_Data_GZ	34.61 56.74	25.61 52.87	96.18 93.98	**97.48** 94.81	29.44 54.74	26.13 48.36

Significance values are in bold.

### Evaluation of the dense block

In this section, we investigate the impact of the modified Dense Block that facilitates feature reuse by establishing connections between the feature maps across network layers. A comparative analysis of our approach, with and without a Dense Block, is presented in Table 4. After incorporating the modified Dense Block into the model architecture, the precision_change, recall_change, precision_no change, F1_score, and Kappa coefficient of the OSCD were improved by 2.88%, 20.61%, 1.02%, 10.87%, and 10.36%, respectively. On the CD_Data_GZ dataset, the precision_change, F1_score, and Kappa coefficient were improved by 15.53%, 14.64%, and 12.59%. The Dense Block leverages feature multiplexing and gradient flow to enhance the network’s ability to learn feature representations in change detection tasks more effectively. This improvement allows the model to identify areas of change more accurately, thereby enhancing precision. Simultaneously, the densely connected design enables the network to capture change information more comprehensively, reducing the likelihood of missed detections. Consequently, both precision and recall are improved, leading to a higher F1_score. The Dense Block thus improves the model’s performance in classification tasks by refining feature representation and gradient flow, which enhances the accuracy of identifying changed and unchanged areas. However, it should be noted that the use of Dense Blocks also increased FPs, resulting in a slight reduction in recall_no_change by 1.1% for OSCD. On the CD_Data_GZ dataset, recall_change, precision_no_change, and recall_no_change decreased by 2.17%, 3.42%, and 1.11%, respectively.

**Table 4:** Evaluation results of architectures with and without dense block.

Models	Dataset	Pre_c (%)	Rec_c (%)	Pre_nc (%)	Rec_nc (%)	F1 (%)	Kappa (%)
Method with Dense Block	OSCD CD_Data_GZ	43.22 **58.29**	57.13 56.03	97.73 94.36	96.09 94.83	49.21 **57.14**	46.18 **51.74**
Method without Dense Block	OSCD CD_Data_GZ	40.34 42.76	36.52 **58.20**	96.71 **97.78**	**97.19** 95.94	38.34 42.50	35.82 39.15

Significance values are in bold.

### Evaluation of the LSTM block

In this section, we investigate the role of the LSTM block in computing the temporal relationship between the bi-temporal images. A comparative analysis of our approach, with and without the LSTM block, is presented in Table 5. On the OSCD dataset, the incorporation of LSTM blocks led to improvements in precision_change, recall_change, precision_no change, F1_score, and Kappa by 0.47%, 9.54%, 0.47%, 4.16%, and 4.96%, respectively. On the CD_Data_GZ dataset, the precision_change, recall_change, F1_score, and Kappa were improved by 6.74%, 19.87%, 7.84%, and 6.03%, respectively. The LSTM’s ability to capture long-term dependencies in data is particularly advantageous for change detection tasks, where changes are often not isolated but correlated with prior and subsequent data points in the time series. Through its unique gating mechanism, LSTM can effectively learn and utilize these long-term dependencies to more accurately identify change points, thereby enhancing precision. This gating mechanism allows LSTM to flexibly adjust the retention of past information, which aids in identifying relevant historical data for current changes, thus improving recall. Consequently, improvements in both precision and recall lead to enhanced F1_score and Kappa values. However, it should be noted that while the LSTM block enhances FP detection capability, it also led to a slight decrease (0.6%) in recall_no_change on the OSCD dataset; on the CD_Data_GZ dataset, precision_no_change and recall_no_change decreased by 2.36% and 3.4%, respectively.

**Table 5:** Evaluation results of architectures with and without LSTM block.

Models	Dataset	Pre_c (%)	Rec_c (%)	Pre_nc (%)	Rec_nc (%)	F1 (%)	Kappa (%)
Method with LSTM block	OSCD CD_Data_GZ	43.22 **58.29**	**57.13** 56.03	**97.73** 94.36	96.09 94.83	49.21 **57.14**	46.18 **51.74**
Method without LSTM block	OSCD CD_Data_GZ	42.75 51.55	47.59 36.16	97.26 96.72	96.69 **98.23**	45.05 49.30	41.22 45.01

Significance values are in bold.

## Conclusion

This paper presents a novel learning framework for urban change detection that integrates a modified Dense Block and an LSTM block within a Bi-Unet architecture. Quantitative and qualitative analyses demonstrated the challenges associated with urban change detection, including significant disparity errors, diverse types of changes, and substantial misalignment between changed and unchanged areas. The experimental results reveal that change detection is influenced by numerous FPs resulting from labeling errors, lighting variations, or other unrelated changes. Our model significantly enhanced performance compared to single-task change-detection frameworks by incorporating bi-temporal tasks. Moreover, both the modified dense and LSTM blocks contribute to improved change detection by employing more stringent formulas. The Dense Block serves as an effective feature extractor that combines identity mapping attributes, depth supervision mechanisms, and diversified depths to facilitate feature reuse throughout the network for more efficient learning processes that aid in reducing FP detection. In contrast, the LSTM block provides an efficient aggregation strategy for skip connections by encoding information from multiple timestamps to generate more meaningful temporal feature vectors. The method proposed in our study demonstrates a substantial reduction in FPs, along with significant improvements in the F1-score and Kappa, thereby successfully identifying labeling errors in both examined datasets.

In future research, we aim to enhance generalization by employing multitask learning with multi-temporal images. The utilization of multitask learning^54^ can improve learning performance through data amplification, attribute selection, representation bias management, and the prevention of overfitting. We integrated multi-temporal images into multiple tasks and simultaneously learned multiple related tasks using multitask learning. During the learning process, a shallow shared representation is employed to facilitate knowledge sharing and complementarity among domains, thereby promoting mutual learning and enhancing generalization.

Determining the initial manifestation of urban change is highly valuable for change detection, and can provide more comprehensive insights into the frequency of urbanization. In this study, we employed an LSTM network to investigate internal activation operations, encompassing both intra-departmental processes and information stored within cell states. As emphasized by Lyu et al.^55^, the cell state effectively preserves crucial information across consecutive time steps, thereby encapsulating alterations in the current sequence image pair. By appropriately fine-tuning our approach, it may be feasible to discern the changes after each temporal increment and accurately track their precise onset.

Finally, we strive to optimize the preservation and shape fidelity of the detected object while minimizing false positives that are in close proximity to true positives.

## Data Availability

Data publicly available in a repository: The dataset of Onera Satellite Change Detection (OSCD) is available at https://ieee-dataport.org/open-access/oscd-onera-satellite-change-detection#files.The dataset of Change Detection Data of Guangzhou (CD_Data_GZ) is available at https://github.com/daifeng2016/Change-Detection-Dataset-for-High-Resolution-Satellite-Imagery. Data available with the paper or supplementary information: The authors declare that the data supporting the findings of this study are available within the paper. Should any raw data files be needed in another format they are available from the corresponding author upon reasonable request. Source data are provided with this paper. Example from: https://www.kaggle.com/code/wanghaiying/urban-change-detection-of-remote-sensing-images.
